# Psoriasis and sexuality: Patients express their feelings

**DOI:** 10.1002/ski2.199

**Published:** 2022-12-28

**Authors:** Romain Salle, Bruno Halioua, Gaëlle Le Fur, Roberte Aubert, Jason Shourick, Charles Taieb

**Affiliations:** ^1^ Department of General and Oncologic Dermatology Université Paris‐Saclay USQY, EA4340‐BECCOH, AP‐HP Ambroise‐Paré hospital Boulogne‐Billancourt France; ^2^ Dermatology Institut Alfred Fournier Paris France; ^3^ Sociologist Paris France; ^4^ President of France Psoriasis [patient association] Paris France; ^5^ Department of Epidemiology, Clinical Epidemiology and Public Health UMR 1027 INSERM‐University of Toulouse III Toulouse University Hospital (CHU) Toulouse France; ^6^ French Society of Human Skin Sciences, [SFSHP] Maison de la dermatologie Paris France; ^7^ Patient Priority Department EMMA Fontenay sous Bois France

## Abstract

**Background:**

In France, psoriasis is a chronic inflammatory skin disease. For several years now, particular attention has been given to the quality of life (Qol) of psoriasis patients. Sexual dysfunction (SD) defined as not wanting or enjoying sexual experience is an important component of Qol. Psoriasis through its physical symptoms and psychological consequences can thus be responsible for SD.

**Method:**

The survey participants were recruited through the national psoriasis dermatitis patient association.

**Result:**

41% (577) patients reported having SD related to their psoriasis. Women reported significantly more SD than men (387 [44.7%] vs. 190 [35%], *p* < 0.001). For 396 (28.1%) patients SD due to their psoriasis manifested as a reduction in the frequency of sexual activity, for 207 (14.7%) as change in their sexual practice, and for 284 (27.4%) as an absence of sexual activity without significant difference between women and men. The main consequence of sexual difficulties for patients was loss of self‐confidence in 627 (44.5%) cases, guilt in 209 (14.8%) cases, couple life impairment in 214 (15.2%) cases, isolation in 260 (18.5%) cases and frustration.

**Discussion:**

In this study investigating SD related to psoriasis, we found that the disease impact sexual relations of 41% of patients of both gender. The main causes of SD were both physical alone and psychological alone, in the same proportions. The physical symptoms of psoriasis are therefore not the only ones responsible for the sexual discomfort reported from patients in their sexual relations. This may be due to a discomfort of the patient or the physician when talking about sexuality because they can't find the terms to talk about this subject often considered taboo and the age or gender difference. This aspect of the disease should be considered by physicians who care for patients with psoriasis, especially dermatologists. Finally, other strategies should be implemented, such as patient talk groups, referral guides or personalised therapeutic education.

## Graphical abstract

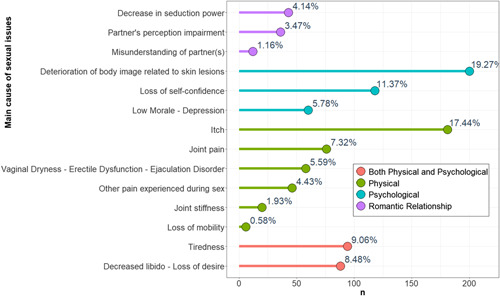

1

**What is already known about this topic?**

- For several years now, particular attention has been given to the quality of life of psoriasis patients.
- Sexual dysfunction defined as not wanting or enjoying sexual experience is an important component of quality of life.
- Psoriasis through its physical symptoms and psychological consequences can thus be responsible for Sexual dysfunction

**What does this study add?**

- The main consequence of sexual difficulties for patients was loss of self‐confidence, guilt, couple life impairment, isolation and frustration

## 2 INTRODUCTION

Psoriasis is a chronic inflammatory skin disease that affects 0.14% to 1.99% of the adult population within different countries.^1^ Psoriasis causes physical symptoms like itch and burning sensations and is associated with psychological disorders such as depression and anxiety.^2^, ^3^ For several years now, particular attention has been given to the quality of life (Qol) of psoriasis patients. This attention has manifested itself with a paradigm shift in the introduction of systemic treatment going from a prescription according to the severity of the disease to one centred on the burden of disease.^4^ Sexual dysfunction (SD) defined as not wanting or enjoying sexual experience is an important component of Qol.^5^ Psoriasis through its physical symptoms and psychological consequences can thus be responsible for SD.^6^ Recent trials have demonstrated that biological therapies improve psoriasis patients' sexual function.^7^, ^8^, ^9^, ^10^, ^11^, ^12^ However, the tools most frequently used in studies to assess sexual dysfunction in psoriasis such as the International Index of Erectile Function‐5 (IIEF), and the Female Sexual Function Index (FSFI) do not allow a global and systemic assessment of the impact of the disease on patients' sexuality of both gender and focus mainly on genital function.^6^ Yet some studies have demonstrated that loss of self‐confidence related to the degradation of body image or the difficulty to reveal their intimacy in front of a sexual partner might actually be an important part of SD in psoriasis patients.^13^ Moreover studies show that sexual function is rarely discussed during medical consultation and that patients struggle to be advised concerning potential solutions.^14^, ^15^

The aim of this survey study is to evaluate SD, its appreciation during medical encounters and patients' opinion on potential solutions to be implemented in clinical practice among male and female psoriasis patients.

## 3 MATERIALS AND METHODS

This was an observational, cross‐sectional, patient driven study.

### 3.1 Study population

The survey participants were recruited between September 2019 and March 2020 through the national psoriasis dermatitis patient association (Association France Psoriasis). The National Association posted an announcement and a link on their website, on social networks and through their newsletter on and a reminder. Respondents who stated that their psoriasis had been diagnosed by a physician were proposed to participate in the study. Inclusion criteria were: (i) being able to understand French language; (ii) having given consent to participate in the study after receiving written information about the study; (iii) being 18 years old or older.

### 3.2 Data collection

Respondents were asked to answer a questionnaire with sociodemographic and personal information (Supplementary Material S1). Questions concerned gender, age category, professional level, relationship status, comorbidities, history of psoriasis and treatment. To limit memory bias, questions about current sexuality were given a 3 month timeframe when possible. To follow a patient driven research methodology closed questions with multiple choice were developed by the patient association and concerned the overall life aspects modified by psoriasis, the way psoriasis impacted their sexual life, the presence or not of SD related to psoriasis or its treatments, the main difficulty that prevented patients from having a satisfying sexual life, the consequences of these difficulties on their sexual life and the management of their SD with their partner. For the main difficulties that prevented patients from having a satisfying sexual life, we have classified them into four main categories: physical issues (itch, joint pain, vaginal dryness‐erectile dysfunction‐ejaculation disorder, other pain experienced during sex, joint stiffness, loss of mobility), psychological issues (deterioration of body image related to skin lesions, loss of self‐confidence, low morale‐depression), both physical and psychological issues (tiredness, decreased libido‐loss of desire) and romantic relationship issues (decreased in seduction power, partner's perception impairment, misunderstanding of partner[s]).

Additional questions were asked about the specialty of their treating physician, whether or not they discuss their sex life with their physician, whether or not they would like to discuss their sex life with their physician, and what tools or services related to psoriasis could help them have a more fulfiling sex life.

### 3.3 Statistical analysis

Categorical values were described as numbers and percentages, and continuous variables as mean and standard deviation. Patients were compared depending on whether they were men or women. Categorical variables were compared using Chi2 test, and continuous variables using *t*‐test.

## 4 RESULTS

### 4.1 Study population

From May to June 2020, 1409 patients answered a questionnaire about their sexual difficulties in relation to their psoriasis. Table 1 reports the main characteristics of the study patients. One hundred and forty‐two (10.1%) patients were under 30 years of age, 379 (26.9%) were between 31 and 45 years of age, 470 (33.4%) were between 46 and 60 years of age and 418 (29.7%) were 61 years of age and older. Eight hundred and sixty six (61.5%) patients were women. The mean disease duration was 23.2 years (± standard deviation 15). In terms of treatment, 862 (61.2%) patients had topical treatment, 148 (10.5%) had phototherapy, 334 (23.7%) had systemic therapy and 314 (22.3%) had biologic therapy. Concerning co‐morbidities, 409 (29%) patients reported an association with psoriatic arthritis, 191 (13.6%) had depression, 180 (12.8%) had obesity, 99 (7%) had diabetes and 88 (6.2%) had cardio‐vascular disorders.

**TABLE 1:** Major baseline demographic and disease characteristics (*n* = 1409)

Characteristic	Total (*n* = 1409)
Missing data (md) = *n* (%)	
Socio‐demographic characteristics	
Age (years)	
<30 years	142 (10.1)
31–45 years	379 (26.9)
46–60 years	470 (33.4)
>61 years	418 (29.7)
Female	866 (61.5)
Disease characteristics	
Mean disease duration (years)	23 (15)
Treatment	
Topical treatment	862 (61.2)
Phototherapy	148 (10.5)
Systemic therapy	334 (23.7)
Biological therapy	314 (22.3)
Comorbidities	
Comorbidities	
Psoriasic arthritis	409 (29)
Obesity	180 (12.8)
Diabetes	99 (7)
Cardio‐vascular disorder	88 (6.2)
Depression	191 (13.6)
Relationship status md = 52 (3.8)	
Single	351 (25.9)
<1 year	56 (4.1)
1–5 years	107 (7.9)
>5 years	188 (13.9)
In couple	1006 (74.1)
<1 year	53 (3.9)
1–5 years	120 (8.8)
5–10 years	117 (8.6)
>10 years	716 (52.8)
Children	1032 (73.2)

*Note*: Mean (SD) for continuous variables and *n* (%) for categorical variables.

### 4.2 SD associated with psoriasis

**FIGURE 1:**
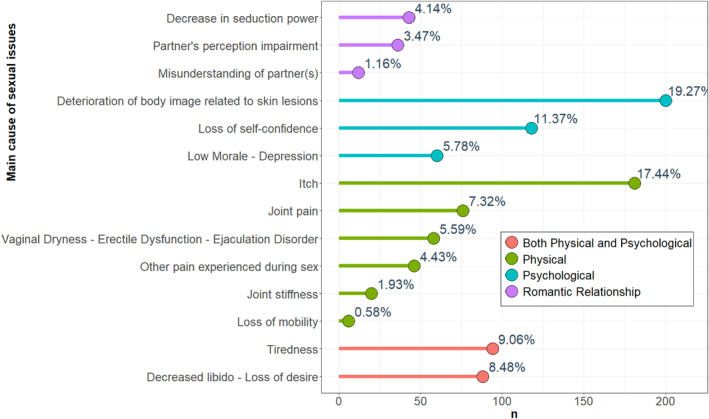
Main cause of sexual issues reported by patients with psoriasis. Of the 1038 answers to the question about the main difficulty that prevented patients from having a satisfying sexual life. Each patient had the possibility to choose up to 3 issues among the different difficulties proposed in the questionnaire

**FIGURE 2:**
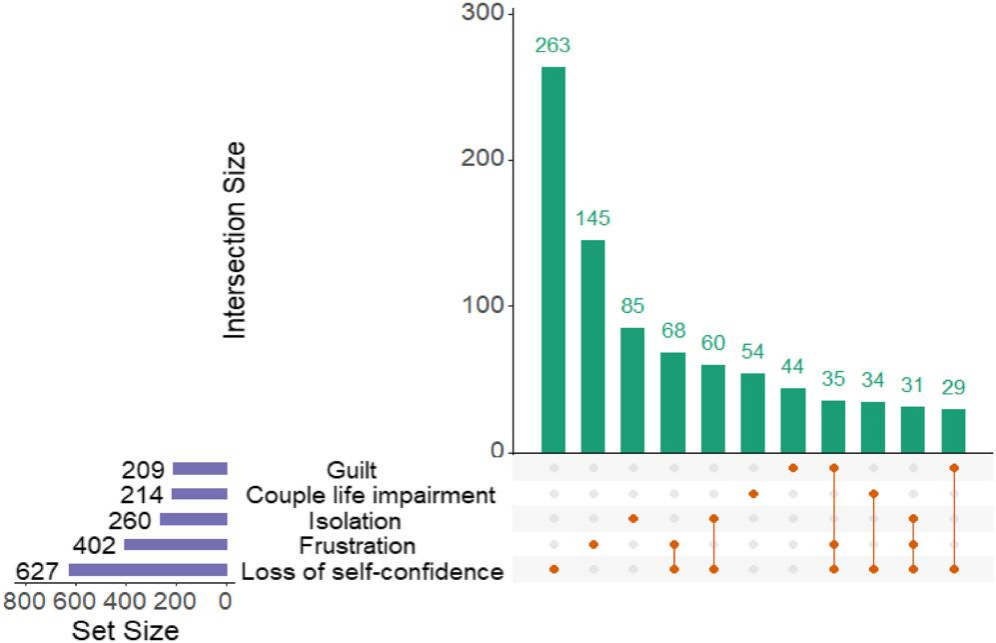
Consequences of sexual dysfunction in psoriasis patients. Upset plot presenting main consequences of psoriasis related sexual dysfunction, Set size (purple barplot) plot presents the prevalence of each cause, combination part reads the following way, the orange dots and connections explain the combination and the green barplot presents combination size

Overall, 577 (41%) patients reported having SD related to their psoriasis. Women reported significantly more SD than men (387 (44.7%) versus 190 (35%), *p* < 0.001) (Table 2). For 396 (28.1%) patients SD due to their psoriasis manifested as a reduction in the frequency of sexual activity, for 207 (14.7%) as change in their sexual practice, and for 284 (27.4%) as an absence of sexual activity without significant difference between women and men. Concerning the main reasons for SD, 1038 responses were available including 387 (37.3%) physical issues alone, 378 (36.4%) psychological issues alone, 182 (17.5%) both physical and psychological issues and 91 (8.8%) romantic relationship issues. The details of the main causes of sexual issues are summarised in Figure 1. No significant difference was found between women and men. The main consequence of sexual difficulties for patients was loss of self‐confidence in 627 (44.5%) cases, guilt in 209 (14.8%) cases, couple life impairment in 214 (15.2%) cases, isolation in 260 (18.5%) cases and frustration in 402 (28.5%) cases **(**Figure 2). Guilt was significantly more reported by women (160 (18.5%) versus 49 (9%), *p* < 0.001), with no significant difference found for other main consequences (Table 2).

**TABLE 2:** Comparison of sexual dysfunction between female and male patients (*n* = 1409)

Characteristic	Female	Male	*p* value
Missing data (md) = *n* (%)	(*n* = 866, 61.5%)	(*n* = 543, 38.5%)	
Comparison of sexual dysfunction			
Sexual dysfunction	387 (44.7)	190 (35)	*p* < 0.001
Reduction in frequency md =	246 (28.4)	150 (27.6)	*p* = 0.699
Modification of sexual practice	130 (15)	77 (14.2)	*p* = 0.692
Absence of sexual activity *md = 371 (26.3)*	180 (28)	104 (26.4)	*p* = 0.616
Comparison of main reasons for sexual dysfunction md = 371 (26.3)			
Physical	251 (39)	136 (34.5)	*p* = 0.062
Psychological	236 (36.6)	142 (36)	
Both physical and psychological	112 (17.4)	70 (17.8)	
Romantic relationship	45 (7)	46 (11.7)	
Comparison of main consequences of sexual dysfunction			
Loss of confidence	398 (46)	229 (42.2)	*p* = 0.169
Guilt	160 (18.5)	49 (9)	*p* < 0.001
Couple life impairment	134 (15.5)	80 (14.7)	*p* = 0.76
Isolation	163 (18.8)	97 (17.9)	*p* = 0.673
Frustration	242 (27.9)	160 (29.5)	*p* = 0.545
Comparison of relationship status and sexual dysfunction impact			
Single	232 (28)	119 (22.5)	*p* < 0.001
Major obstacle to the search for a new partner	216 (24.9)	142 (26.2)	*p* = 0.545
In couple	595 (72)	411 (77.5)	*p* < 0.001
Felt good in their body	667 (77)	426 (78.5)	*p* = 0.555
Could express their issues with their partner	718 (82.9)	449 (82.7)	*p* = 0.942
Felt understood by their partner	729 (84.2)	473 (87.1)	*p* = 0.142
Could find a solution to their issues with their partner	720 (83.1)	466 (85.8)	*p* = 0.202

*Note*: *n* (%) for categorical variables.

### 4.3 Managing SD with one's partner

**FIGURE 3:**
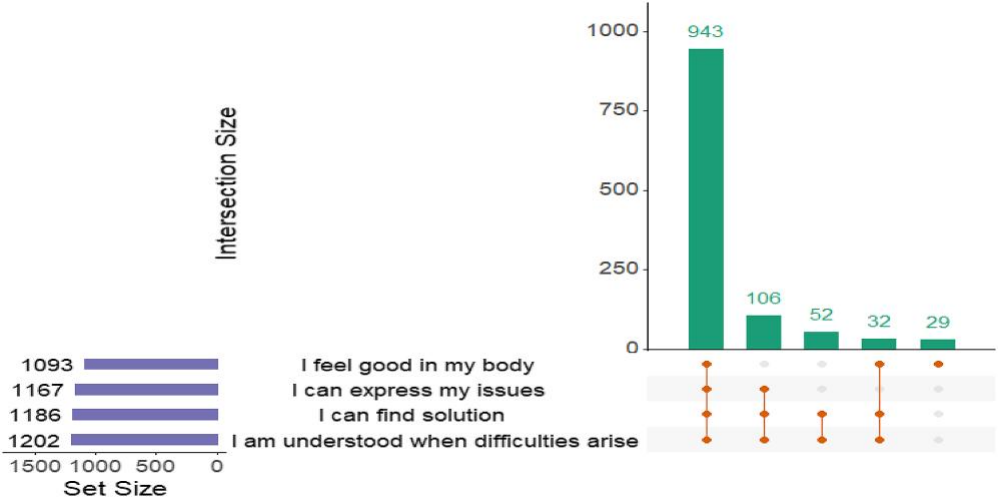
Relationship with partners in psoriasis patients. Upset plot presenting relation with partner concerning psoriasis related sexual dysfunction, Set size (purple barplot) plot presents the prevalence of each partners related efffect, combination part reads the following way, the orange dots and connections explain the combination and the green barplot presents combination size

Regarding the family situation, 351 (25.9%) patients were single (188 (13.9%) for more than 5 years), 1006 (74.1%) patients were in couples (716 (52.8%) more than 10 years) and 1032 (73.2%) patients had children. Regarding their relationship with their partner, 1093 (77.6%) patients felt good in their body, 1167 (82.8%) patients could express their issues, 1202 (85.3%) patients felt understood by their partner and 1186 (84.2%) patients could find a solution to their issues (Figure 3). For single patients, psoriasis was considered a major obstacle for finding a new partner in 358 (25.4%) cases. Women were significantly more likely to be single, but there was no difference in terms of difficulty in finding a new partner. For patients in couples, there was no difference between sexes in the management of sexual dysfunction with their partner (Table 2).

### 4.4 SD care management

Overall, 561 (39.8%) patients were taken in charge by a dermatologist. Other physicians involved were general practitioners (14.5%) and rheumatologists (8.6%). Among patients, 783 (81.3%) never had a conversation about sex with their physician. If a conversation took place, it was started in 97 (10.1%) cases by the patient, in 75 cases (7.8%) by the doctor and 7 (0.7%) cases by both. Four hundred and one (28.5%) patients would be more interested in discussing their SD with their physicians. The means to manage their psoriasis SD proposed by patients were a collection of stories or advice from other patients (28.3%), a support compiling general recommendations from various qualified professionals (25%), a forum for anonymous exchanges with other patients (17.9%), individual exchange times with a sexologist (12%), a discussion groups led by a psychologist or sexologist (12.4%) and guide of positions adapted according to painful areas (4.5%).

## 5 DISCUSSION

In this study investigating SD related to psoriasis, we found that the disease impact sexual relations of 41% of patients of both gender. This result is consistent with several other studies.^16^, ^17^, ^18^ Comparing the two genders, we also find that sexual disorders are more often present in women. This gender difference has been observed before, where women with psoriasis are at greater risk of SD than man.^6^, ^19^ These new data seem to be explained by the fact that women are more impacted by the negative psychological and sexual consequences of psoriasis as well as the degradation of body image which induces an alteration of the capacities of seduction.^17^

In our study, the main causes of SD were both physical alone and psychological alone, in the same proportions. The physical symptoms of psoriasis are therefore not the only ones responsible for the sexual discomfort reported from patients in their sexual relations. The psychological aspect thus seems to become predominant in the sexual life of psoriasis patients, for whom skin lesions are more disabling in their appearance than in their physical impact. This observation also argues that the severity of the disease is not necessarily related to the intensity of psychological disorders in psoriasis patients.^20^ In some patients, deterioration in body image related to the skin lesion and loss of self‐confidence was reported as difficulties related to their sexual life which is consistent with a study on the effect of body image on SD in psoriasis.^13^ In addition, the multiplicity of Qol domains affected by psoriasis confirms their concomitant influences on patients' sexual functions and their mutual influences on each other.^21^

The SD reported by the patients in this study also have psychological consequences of its own, including loss of self‐confidence, frustration, isolation and guilt, which is described more often by women. In addition, 14% of patients reported suffering from depression in our study, a similar proportion found in other studies.^2^ However, depressive disorders in psoriasis are described as both a cause and a consequence of SD.^8^, ^16^, ^17^, ^22^, ^23^ Thus, psoriasis patients suffering from SD find themselves in a vicious circle where the psychological impact of these disorders leads to new psychological complications which in turn will further handicap patients in their sexual lives.

When we look at the consequences of SD within their relationships, it seems that this is well managed by patients in couples (in both genders), especially through discussion with their partners. The fact that patients can exchange and find solutions with their partners is a positive point. Indeed, psoriasis can also have a psychological impact on the partner, sometimes greater than the patient himself.^24^ This can then deteriorate family life, worsen the patient's quality of life and thus lead to additional psychological distress. On the contrary, psoriasis seems to be a handicap in the search of new partners by single patients who report it as a major obstacle in 25% of cases and equally between male and female. This notion is also found in other skin diseases such as vitiligo^25^ and can be explained by the degradation of body image or the loss of self‐confidence of the patient secondary to the stigmatising nature of these skin lesions. This difficulty in being able to look for a new partner can then aggravate the patient's feelings of isolation or frustration and cause even greater psychological distress. In this sense, special attention should be given to the single patient experiencing these relational difficulties so that systemic treatment can be provided early in the history of the disease. Especially since the patient's psychological distress is not always related to the extent of skin lesions or clinical severity.^20^

In the meantime, there is likely a lack of care for SD by physicians that patients during clinical encounters for psoriasis. First of all, there is a miscommunication on the part of patients and physicians about SD. Similarly to other studies,^26^ most patients (81%) never had a conversation with their physician about their SD and when they did it was more often started by patients than physician.

This may be due to a discomfort of the patient or the physician when talking about sexuality because they can't find the terms to talk about this subject often considered taboo and the age or gender difference between patients and their physician.^26^, ^27^ However it may also be due to a lack of information about the link between psoriasis and SD and that some treatments of psoriasis can improve SD management.

Contrary to other pathologies such as diabetes where sexuality is more easily addressed by general practitioners,^28^ it is also likely that physicians caring for psoriatic patients may not always be aware that SD is common in this condition. Furthermore, physicians should be aware that SD is a real concern for patients which can handicap their relationships and be responsible for psychological distress. Particularly given the fact that 29% of patients in our study would be more interested in discussing their SD with their physicians. SD should therefore be routinely discussed during consultations with psoriasis patients in order to anticipate their needs and expectations in their care.

Moreover, our study also explores the different media through which patients would like to be informed and manage their sexual problems related to psoriasis. To begin, patients highlighted the need to feel more supported and share with other patients, while still favouring anonymous and online possibilities. This reflects the need for patients to feel more accompanied and realize how common is their SD among other psoriasis patients. Patients also favoured the help of practical guides such as a support compiling general recommendations or sexual positions adapted according to the painful areas whether on paper or digital formats. Finally, within their care pathway, patients also request time for exchange and discussion, with physicians but also with other professions, more specifically with psychologists, or sexologists who are specialised in the management of SD. It would therefore be interesting to integrate the patient in the management of his SD through personalised therapeutic education and to have a multidisciplinary vision of the Qol in psoriasis in terms of sexuality as it is already the case in other diseases such as HIV.^29^

Our study had several limitations. First, the format of the questionnaire may have led to a memorisation bias because patients who had previously identified sexual difficulties or problems in their relationships were more able to recall SD. To minimise this bias we gave a 3 month timeframe whenever it was possible. It is also difficult to assess SD in single patients because practice and expectations differ from one person to another. For example, one party may not be sexually active and therefore may not be concerned with an important amount of the questionnaire and even on an online questionnaire might still be taboo for some. However using the online questionnaire format and having questions designed by the patient themselves minimised the risk to have inappropriate items.

Second, our study did not specifically assess physical problems related to sexual disorders such as ED, vaginal dryness or ejaculation disorders because these three items were combined in the questionnaire. The majority of studies focussing on SD in psoriasis take these factors into account,^6^ especially as ED is a major component of sexual dysfunction in psoriasis, often related to the patient's psychological state.^30^ However the aim of our study was to more specifically assess the psychological and relational mechanisms responsible for SD in psoriasis as well as how patients feel about their partners and physicians.

Similarly, the locations of psoriasis skin lesions were not asked for in the questionnaire despite the involvement of certain locations, particularly genital psoriasis, in SD and impaired Qol.^31^ Moreover, genital psoriasis is often under‐diagnosed in consultation mainly due to patient discomfort or a lack of attention from the physician,^32^ which in light of our study could be related to the miscommunication on SD.

Finally, since this questionnaire was not conducted by a physician, it was not possible for us to determine the severity of patients' psoriasis as well as the accuracy of their medical history or treatments. Consequently, we cannot determine whether the intensity of the SD reported by patients is related to the severity of their dermatological condition nor can we determine whether the treatments received by patients, and more specifically biological therapies, have led to an improvement as demonstrated in previous studies.^7^, ^8^, ^9^, ^10^, ^11^, ^12^ It would therefore be interesting to evaluate whether the improvement of psoriasis sexual disorders following treatment could also improve the psychological and relational consequences, in association with the use of patient guides and multidisciplinary management with sexologists and psychologists.

In conclusion this study shows that psoriasis can be responsible for SD in patients because of physical suffering but also resulting from possible psychological impairment. This aspect of the disease should be considered by physicians who care for patients with psoriasis, especially dermatologists. Finally, other strategies should be implemented, such as patient talk groups, referral guides or personalised therapeutic education.

## CONFLICTS OF INTEREST

The authors declare that there is no conflict of interest.

## AUTHOR CONTRIBUTIONS

**Romain Salle:** Validation (Equal); Writing – original draft (Equal); Writing – review & editing (Equal). **Bruno Halioua:** Methodology (Equal); Validation (Equal); Writing – original draft (Equal). **Gaelle Le Fur:** Conceptualisation (Equal); Supervision (Equal); Writing – original draft (Equal). **Roberte Aubert:** Conceptualisation (Equal); Project administration (Equal); Validation (Equal); Writing – original draft (Equal). **Jason Shourick:** Conceptualisation (Equal); Project administration (Equal); Validation (Equal); Writing – original draft (Equal). **Charles Taieb:** Conceptualisation (Equal); Data curation (Equal); Methodology (Lead); Project administration (Lead); Supervision (Equal); Visualisation (Equal); Writing – original draft (Equal); Writing – review & editing (Equal).

## ETHICS STATEMENT

Not applicable.

## Supporting information

Supporting Information S1

## Data Availability

The data that support the findings of this study are available from the corresponding author upon reasonable request.
