# Supplementary material for: Psoriasis and sexuality: Patients express their feelings
Source: Skin Health Dis. 2022 Dec 28;3(3):e199. doi: 10.1002/ski2.199 (PMC10233079; doi:10.1002/ski2.199)
Supplement: Supplementary file 1 — Supporting Information S1 [file SKI2-3-e199-s001.pdf]

Bien-être, estime de soi, relation avec l’autre, mais aussi conception… L’épanouissement sexuel fait partie intégrante de la qualité de vie.

Pourtant, lorsqu’on souffre d’un psoriasis, la vie intime peut être entravée, engendrer des craintes ou des frustrations dont il n’est pas toujours facile de parler et qui sont trop peu souvent prises en compte…

Grâce à cette enquête totalement anonyme, réalisée avec le soutien institutionnel d’UCB Pharma, nous vous donnons la parole : quelles sont les répercussions de votre psoriasis sur votre vie intime ? Quelles difficultés rencontrez-vous ? Quelles attentes avez-vous ?

Partagez votre expérience et votre ressenti, ce questionnaire ne vous prendra qu’une dizaine de minutes !

1.
Dans quelle région française résidez-vous ?

2.
Quel est votre sexe ?

- Homme
- Femme

3.
Quelle est votre tranche d'âge ?
*

- 18 ans ou moins
- 19-30 ans
- 31-45 ans
- 46-60 ans
- 61 ans ou plus

4.
Quel est votre plus haut niveau de formation ?
*

- Primaire
- Secondaire (sans le BAC)
- Secondaire (avec le BAC)
- Post-secondaire non-supérieur
- Supérieur de cycle court (ex. : DUT, DEUG, BTS...)
- Licence
- Master
- Doctorat

○ Autre, merci de préciser ... [ ]

5.
Dans quelle catégorie socio-professionnelle vous situez-vous?
*

- ○ Cadres et profession intellectuelle supérieure
- ○ Profession intérmediaire, cadre moyen
- ○ Etudiant
- ○ Ouvrier qualifié
- ○ Artisan, commerçant, chef d'entreprise, profession libérale
- ○ Employé et personnel de service
- ○ Sans emploi
- ○ Main d'oeuvre et ouvrier specialisé
- ○ Agriculteur, exploitant
- ○ Retraité

6.
Êtes-vous atteint(e) d’un psoriasis ?

- ○ Oui
- ○ Non

7.
Actuellement, quelle est votre situation de couple ?
*
(Pour faciliter l’analyse de l’enquête, la question ne prend pas en compte les statuts de veuf(ve) ou divorcé(e) : Si vous êtes veuf(ve) ou divorcé(e) et que vous ne vous êtes pas remis(e) en couple , merci de c

- ○ En couple depuis moins d’1 an
- ○ En couple depuis 1 à 5 ans
- ○ En couple depuis 5 à 10 ans
- ○ En couple depuis 10 ans et plus
- ○ Célibataire/seul(e) depuis moins d’1 an
- ○ Célibataire/seul(e) depuis depuis 1 à 5 ans
- ○ Célibataire / seul(e) depuis plus de 5 ans
- ○ Non concerné(e)

8.
Avez-vous eu un ou des enfants?

- ○ Oui
- ○ Non

9.

Quel âge a ou ont-il (s) ?
*
(Facultatif / Plusieurs réponses possibles)

- ☐ Moins de 5 ans
- ☐ 5 à 10 ans
- ☐ 11 à 20 ans
- ☐ 20 ans et plus

10.
Votre Psoriasis constitue-t-il un frein majeur dans la recherche d'un nouveau partenaire ?
*

- ○ Oui
- ○ Non
- ○ Non concerné (e)

Psoriasis et vie intime

Maladies et traitements

11.
Depuis combien d'années souffrez-vous de votre Psoriasis ?

12.
Par quelle(s) autre(s) maladie(s) êtes-vous concerné(e) ?
*
(Facultatif / Plusieurs réponses possibles)

- ☐ Rhumatisme psoriasique
- ☐ Maladie cardiovasculaire
- ☐ Diabète
- ☐ Maladie de Crohn ou RCH
- ☐ Ostéoporose
- ☐ Lupus
- ☐ Aucune de ces pathologies
- ☐ Obésité
- ☐ Dépression
- ☐ Autre, merci de préciser ... ________

13.
Pour votre psoriasis, êtes-vous traité(e) par :
*

	Oui	Non	Je ne sais pas
Traitement(s) dermocorticoïde(s) local-aux (sous forme de crème, pommade, gel ou film), avec ou non association de dérivés de vitamine A ou D	☐	☐	☐
Photothérapie (par rayons UVA ou UVB en cabinet de dermatologie)	☐	☐	☐
Traitement(s) de fond systémique(s) (médicaments par voie orale)	☐	☐	☐
Traitement(s) de fond biologique(s) (sous forme d'injections sous cutanées ou intraveineuses)	☐	☐	☐
Cures thermales (généralement prescrites en complément d'autres traitements)	☐	☐	☐

14.
Il y-a-t-il un (ou des) traitements que vous prenez et qui n'est (ou ne sont pas)dans la liste ?

○ Oui
○ Non

15.
Pouriez-vous préciser le (ou les) traitement (s) qui n'était (ent) pas listé(s)?

16.
Sur une échelle de 0 à 10, diriez-vous qu'avec vos traitements actuels votre psoriasis est bien contrôlé ?
0 pour « Pas du tout »
10 pour « totalement

☑ NSP (Ne Sais Pas)

Psoriasis et vie intime & Expérience et ressenti

17.
Parmi les propositions ci-dessous, sur quel(s) aspect(s) de votre vie la maladie a-t-elle les répercussions les plus négatives ?
*
(3 réponses maximum)

- ☐ Votre vie professionnelle
- ☐ Votre vie sociale
- ☐ Votre pratique sportive
- ☐ Votre vie familiale
- ☐ Votre vie de couple

- [ ] Votre capacité à vous projeter
- [ ] Votre moral
- [ ] Vos loisirs
- [ ] La réalisation des tâches du quotidien

Max 3

18.
De quelle manière votre psoriasis a-t-il impacté votre vie sexuelle ?

- [ ] Il vous a amené(e) à réduire la fréquence de vos rapports sexuels
- [ ] Il vous a obligé(e) à modifier vos pratiques sexuelles
- [ ] Ni l'un, ni l'autre
- [ ] Vous n'êtes pas concerné(e)

19.
Avez-vous eu un ou des rapports sexuel au cours des trois derniers mois?
*

- ○ Oui
- ○ Non

20.
Si vous n’avez pas eu de rapport sexuel au cours des trois derniers mois, est-ce lié à votre psoriasis ?
*

- ○ Oui
- ○ Non

21.
Au cours des trois derniers mois, combien de rapports sexuels avez-vous eus ?
*

22.
Aviez-vous déjà cessé toute activité sexuelle lorsque votre psoriasis s'est déclaré ?
*

- ○ Oui
- ○ Non

23.
Rencontrez-vous des difficultés liées à votre psoriasis ou à vos traitements qui nuisent à votre vie sexuelle ?
*

(Par exemple : fatigue, douleurs articulaires, autres douleurs ressenties pendant l’acte sexuel, perte de mobilité, raideurs articulaires, baisse de libido, sècheresse vaginale ou dysfonction érectile, dégradation de l&am

- Oui
- Non

24.
Parmi ces mêmes propositions, quelle est/était LA principale difficulté liée à votre psoriasis et/ou à vos traitements qui vous empêche d'avoir une vie sexuelle totalement épanouie ?
*
(Facultatif / Plusieurs réponses possibles)

- Autres douleurs ressenties pendant l'acte sexuel
- Incompréhension du/des partenaire(s) / Peur du partenaire de faire mal
- Dévalorisation vis-à-vis du partenaire
- Altération du toucher due aux plaques de psoriasis
- Douleurs articulaires
- Baisse de la libido / Perte de désir
- Sècheresse vaginale / Dysfonction érectile / Trouble de l'éjaculation
- Dégradation de l'image corporelle liée aux atteintes cutanées
- Baisse de moral / Déprime
- Diminution du pouvoir de séduction
- Démangeaisons
- Perte de confiance en vous
- Raideurs articulaires
- Fatigue

Max 3

25.
Parmi ces mêmes propositions, quelle est/était **LA** principale difficulté, liée à votre psoriasis et/ou à vos traitements, qui vous empêche d'avoir une vie sexuelle totalement épanouie ?

*
(Une seule réponse possible)

- Démangeaisons
- Fatigue
- Douleurs articulaires
- Autres douleurs ressenties pendant l'acte sexuel
- Perte de mobilité
- Raideurs articulaires
- Baisse de la libido / Perte de désir
- Sècheresse vaginale / Dysfonction érectile / Trouble de l'éjaculation
- Dégradation de l'image corporelle liée aux atteintes cutanées
- Diminution du pouvoir de séduction
- Perte de confiance en vous
- Baisse de moral / Déprime
- Incompréhension du/des partenaire(s)

- ○ Dévalorisation vis-à-vis du partenaire

26.
Attribuez-vous les difficultés que vous rencontrez dans votre vie sexuelle :
*

- ○ Principalement à votre psoriasis
- ○ Principalement au(x) traitement(s)
- ○ Vous ne savez pas

27.
Quelles sont les conséquences de ces difficultés ?
*
(Facultatif / Plusieurs réponses possibles)

- ☐ Dégradation de la relation de couple
- ☐ Frustration
- ☐ Culpabilité
- ☐ Perte d’estime de soi
- ☐ Isolement
- ☐ Difficultés à concevoir un enfant

28.
Globalement, diriez-vous que vous avez une vie sexuelle épanouie ?
Déplacez le curseur pour répondre.
0 pour « Pas du tout »
10 pour « totalement

- ☑ NSP (Ne Sais Pas)

29.
Pensez-vous que, sans la maladie, le score précédent serait :

- ○ Identique
- ○ Non, inférieur
- ○ Non, superieur

30.
Selon vous de combien de point(s) il serait inférieur?
*

31.
Selon vous de combien de point(s) il serait supérieur ?
*

Psoriasis et vie intime & Avec votre partenaire

32.
Diriez-vous qu'avec votre(vos) partenaire(s), dans l'intimité sexuelle, vous vous sentez :
*

	Oui	Non	Ça dépend	Pas concerné(e)
Bien dans votre corps	○	○	○	○
À l'aise pour exprimer vos éventuelles difficultés	○	○	○	○
• Suffisamment compris(e) lorsque vous évoquez vos difficultés	○	○	○	○
• Soutenu(e) pour trouver ensemble des solutions ou adaptations	○	○	○	○

Psoriasis et vie intime & Dialogue avec votre professionnel de santé

33.
Quel est le professionnel de santé qui orchestre la prise en charge de votre psoriasis ?
*

- ○ Dermatologue libéral
- ○ Dermatologue hospitalier
- ○ Rhumatologue libéral
- ○ Rhumatologue hospitalier
- ○ Médecin généraliste
- ○ Médecin interniste
- ○ Médecine alternative
- ○ Autre, merci de preciser ____

34.
Avez-vous déjà échangé, avec lui/elle, au sujet des répercussions de votre psoriasis sur votre sexualité ?
*

- ☐ Oui, c'est lui/elle qui a abordé le sujet
- ☐ Oui, c'est vous qui avez abordé le sujet
- ☐ Non, vous n'avez jamais eu d'échange à ce sujet

35.
Parmi les autres professionnels suivants, avec le(s)quel(s) avez-vous déjà pu échanger sur les répercussions de votre psoriasis sur votre vie sexuelle ?

*
(Facultatif / Plusieurs réponses possibles)

- Médecin généraliste
- Gynécologue
- Urologue
- Psychologue
- Sexologue
- Infirmier(e)
- Association de patients
- Autre, merci de preciser

36.
Souhaiteriez-vous avoir davantage la possibilité d'échanger avec les professionnels de santé, au sujet des répercussions de votre psoriasis sur votre sexualité ?

- Oui
- Non

37.
De la part de quel(s) professionnel(s) attendez-vous davantage de dialogue autour des répercussions de votre psoriasis sur votre sexualité ?
*

- Médecin généraliste
- Gynécologue
- Urologue
- Psychologue
- Sexologue
- Infirmier(e)
- Association de patients
- Dermatologue
- Rhumatologue
- Autre, merci de preciser

38.
Essayez-vous de concevoir un enfant OU avez-vous essayé de concevoir un enfant alors que vous souffriez déjà d'un psoriasis ?
*

- Oui
- Non

39.

Parmi les professionnels suivants, avec le(s)quel(s) avez-vous pu échanger sur les répercussions de votre psoriasis sur la conception d'un enfant ?
*
(Facultatif / Plusieurs réponses possibles)

- Médecin généraliste
- Gynécologue
- Urologue
- Psychologue
- Sexologue
- Infirmier(e)
- Association de patients
- Dermatologue
- Rhumatologue
- Autre, merci de preciser

40.
Souhaitez-vous (ou auriez-vous souhaité) avoir davantage la possibilité d'échanger avec les professionnels de santé, au sujet des répercussions de votre psoriasis sur la conception d'un enfant
*

- Oui
- Non

41.
De la part de quel(s) professionnel(s) attendez-vous (ou auriez-vous attendu) davantage de dialogue autour des répercussions de votre psoriasis sur la conception d'un enfant ?
*
(Facultatif / Plusieurs réponses possibles)

- Médecin généraliste
- Gynécologue
- Urologue
- Psychologue
- Sexologue
- Infirmier(e)
- Association de patients
- Dermatologue
- Rhumatologue
- Autre, merci de preciser

Psoriasis et vie intime & Outils et services

42.
Quels outils ou services de France Psoriasis pourraient vous aider à avoir une vie intime plus épanouie ?
(Facultatif / Plusieurs réponses possibles)

- Des temps d’échange individuels avec un(e) sexologue
- Un recueil de témoignages, conseils, trucs et astuces de malades
- Des groupes de parole avec d’autres malades, animés par un(e) psychologue et/ou un(e) sexologue
- Un forum permettant d’échanger de manière anonyme avec d’autres malades
- Un répertoire des positions adaptées en fonction des zones douloureuses
- Un support compilant des recommandations générales de la part des différents professionnels qualifiés
- Autre, merci de preciser
